# Evaluation of a Combined Live Attenuated Vaccine against Lumpy Skin Disease, Contagious Bovine Pleuropneumonia and Rift Valley Fever

**DOI:** 10.3390/vaccines12030302

**Published:** 2024-03-13

**Authors:** Zohra Bamouh, Amal Elarkam, Soufiane Elmejdoub, Jihane Hamdi, Zineb Boumart, Greg Smith, Matthew Suderman, Mahder Teffera, Hezron Wesonga, Stephen Wilson, Douglas M. Watts, Shawn Babiuk, Brad Pickering, Mehdi Elharrak

**Affiliations:** 1 Research and Development, MCI Santé Animale, Lot. 157, Z. I., Sud-Ouest (ERAC) B.P: 278, Mohammedia 28810, Morocco; a.elarkam@mci-santeanimale.com (A.E.); s.elmejdoub@mci-santeanimale.com (S.E.); jihaneh.hamdi@gmail.com (J.H.); z.boumart2@gmail.com (Z.B.); m.elharrak@mci-santeanimale.com (M.E.); 2 National Centre for Foreign Animal Disease, Canadian Food Inspection Agency, Winnipeg, MB R3E EM4, Canadamatthew.suderman@inspection.gc.ca (M.S.); mahder.teffera@inpection.gc.ca (M.T.); shawn.babiuk@inspection.gc.ca (S.B.); bradley.pickering@inspection.ca (B.P.); 3 Kenya Agricultural and Livestock Research Organization (KALRO), Kaptagat Rd., Loresho, P.O. Box 57811, Nairobi 00200, Kenya; hezron.wesonga@gmail.com; 4 GALVmed, Pentlands Science Park, Edinburgh EH26 0PZ, UK; steve.wilson@galvmed.org; 5 Department of Biological Sciences, University of Texas at El Paso, El Paso, TX 79968, USA; dwatts2@utep.edu

**Keywords:** cattle, immunogenicity, efficacy, antibody, vaccine, lumpy skin disease, contagious bovine pleuropneumonia, Rift Valley fever

## Abstract

The use of effective vaccines is among the most important strategies for the prevention and progressive control of transboundary infectious animal diseases. However, the use of vaccine is often impeded by the cost, a lack of cold chains and other factors. In resource-limited countries in Africa, one approach to improve coverage and reduce cost is to vaccinate against multiple diseases using combined vaccines. Therefore, the objective of this study was to evaluate a combined vaccine for the prevention and control of Lumpy Skin Disease (LSD), Contagious Bovine Pleuropneumonia (CBPP) and Rift Valley fever (RVF). The LSD and CBPP were formulated as a combined vaccine, and the RVF was formulated separately as live attenuated vaccines. These consisted of a Mycoplasma MmmSC T1/44 strain that was propagated in Hayflick-modified medium, RVF virus vaccine, C13T strain prepared in African green monkey cells (Vero), and the LSDV Neethling vaccine strain prepared in primary testis cells. The vaccines were tested for safety via the subcutaneous route in both young calves and pregnant heifers with no side effect, abortion or teratogenicity. The vaccination of calves induced seroconversions for all three vaccines starting from day 7 post-vaccination (PV), with rates of 50% for LSD, 70% for CBPP and 100% for RVF, or rates similar to those obtained with monovalent vaccines. The challenge of cattle vaccinated with the LSD/CBPP and the RVF vaccine afforded full protection against virulent strains of LSDV and RVFV. A satisfactory level of protection against a CBPP challenge was observed, with 50% of protection at 6 months and 81% at 13 months PV. A mass vaccination trial was performed in four regions of Burkina Faso that confirmed safety and specific antibody responses induced by the vaccines. The multivalent LSD/CBPP+RVF vaccine provides a novel and beneficial approach to the control of the three diseases through one intervention and, therefore, reduces the cost and improves vaccination coverage.

## 1. Background

Cattle are critically important as daily sources of food, nutrition and income, and as a source of nitrogen-rich manure for replenishing soils [1]. African cattle are treasured assets for an estimated 800 million livestock keepers across the continent, and they play a major role in socioeconomic development and remain major socio-cultural assets in many African societies [2,3]. In sub-Saharan countries, livestock production accounts for 25% of economic activity and employs about 175 million people directly, with approximately 80% of these animals held by small farm owners who depend on livestock for their livelihoods [4,5]. Livestock diseases negatively affect the national economy, and some of these diseases either threaten or harm human health [6,7,8,9]. Contagious Bovine Pleuropneumonia (CBPP) is a respiratory disease of cattle in Africa, which has serious economic and trade implications across the word [10,11,12]. The control of CBPP is a challenge in Africa because governments do not have the resources to compensate farmers using a stamping-out policy [13]. In addition, other infectious diseases co-infect cattle and require frequent vaccination, thus increasing the costs of control [14,15].

Vaccination remains one of the most important tools for the control of diseases [16,17,18,19]. CBPP has been eradicated from many countries mostly through the stamping-out strategy or vaccination campaigns [13,20,21,22,23]. In contrast, LSD was eradicated in Eastern Europe through vaccination [24]. Although many diseases can be controlled through vaccination where vaccines are available, the vaccination campaign cost and cold-chain delivery impede their widespread use, especially in isolated rural areas [19,25,26]. The right choice for Africa is, therefore, to vaccinate against multiple diseases with combined vaccines to improve coverage and to save cost [23,27,28,29].

A combined LSD/CBPP vaccine was developed to protect cattle against both diseases with a single vaccination [30]. The vaccine was tested in cattle and found to be safe and effective [30]. Rift Valley fever (RVF) is of particular concern because it is a viral disease of livestock that poses a serious public health risk [31,32,33,34]. It is endemic in large parts of Africa, occurring as epizootics/epidemics at irregular intervals [25,34,35]. An effective way to establish solid herd immunity is through regular vaccination, but this is not widely practiced due to irregular and long intervals between epizootic/epidemic periods [36,37,38]. The lack of a visible impact and associated costs during inter-epizootic/epidemic periods is a deterrent in implementing RVF vaccination, exposing highly susceptible animals when outbreaks emerge [16,39,40]. As an effort to overcome the obstacles for using an RVF vaccine, the addition of the RVF vaccine along with the combination vaccine that farmers regularly use (such as LSD or CBPP) could possibly build the necessary RVF herd immunity to protect susceptible livestock against unexpected epizootics/epidemics [41].

Therefore, the objective of this study was to evaluate the safety, efficacy and duration of immunity of a combined live attenuated LSD/CBPP+RVF vaccine for the prevention of these diseases among cattle at a laboratory level and in a large-scale vaccination trial under field conditions.

## 2. Materials and Methods

### 2.1. Live Pathogens Preparation

Live attenuated Mycoplasma MmmSC T1/44 strain (CIRAD AF262936) was prepared as described by Safini et al., 2022 [30]. Briefly, Mycoplasma was cultured in Hayflick-modified medium, supplemented by 10% of equine serum and incubated for 24 h at 37 °C with agitation (100 rpm). The harvested bacterial suspension was inoculated at a ratio of 10% in a bioreactor containing the same media and incubated for 36 h at 37 °C, pH 7.2, with agitation speed of 100 rpm.

Viral strain RVFV C13T was propagated on Vero cells (African green monkey kidney cells, ATCC No. CCL-81) and LSDV Neethling (Pirbright Institute, Surrey, UK) vaccine strain on primary testis cells. Cells were maintained in Dulbecco’s Modified Eagle’s Medium (DMEM) (Thermo Fisher Scientific, New York, United States) with 10% irradiated Fetal Bovine Serum (FBS) (Wisent bioproducts, Quebec, Canada). The medium was removed from the cell confluence and replaced by a viral inoculum at 0.01 Multiplicity of Infection (MOI). After 1 h of incubation at 37 °C, the inoculum was removed, replaced with DMEM with 1% FBS and incubated for 5 days at 35 °C for LSDV and 3 days at 37 °C for RVFV until the cytopathogenic effect (CPE) became apparent. Samples were obtained to determine infectivity titers, purity and identity using PCR for each virus.

### 2.2. Vaccine Preparation

The combined LSD/CBPP vaccine and the monovalent RVF vaccine was prepared separately by adding antigen suspensions to an equal volume of a stabilizer (4% peptone, 8% sucrose and 2% glutamate) and then freeze-dried in an LSI lyodryer (Lyogroup.in, Hyderabad, India) [30]. Vaccines were tested for sterility, identity, purity and infectious titers.

### 2.3. Animals’ Vaccination

#### 2.3.1. Ethics

The animal experiments were conducted at MCI Santé Animale in accordance with Animal Research Reporting of In Vivo Experiments (ARRIVE) guidelines (https://arriveguidelines.org/ (accessed on 17 May 2019), and the handling of experimental animals was performed as described in a protocol approved by MCI Santé Animale Ethic Committee for Animal Experiment Protocol number MCI-R70A1076.

The animal experiments at the National Centre for Foreign Animal Disease were conducted under the approval of the Canadian Science Center for Human and Animal Health, Animal Care Committee, according to the guidelines of the Canadian Council on Animal Care. Animals were housed in groups to allow for normal social interaction. The Animal Technicians care staff were trained in daily animal handling; husbandry; recognition of signs of pain, distress and disease; and the ethics of the use of animals in research.

#### 2.3.2. Safety in Calves

Twenty-four Holstein cross breed calves, 4 months’ old, were housed in an insect-proof building for two weeks to acclimatize and were fed a complete balanced diet and water ad libitum. Calves were monitored daily for body temperature and general health conditions prior to use in the experiments. Calves were randomly selected and were divided into 3 groups (G) of 8 animals each.

Group (GI) was vaccinated with LSD/CBPP+RVF vaccine with a dose of 10^4.5^ Tissue Culture Infectious Dose _50_ (TCID_50_) for Lumpy Skin Disease virus (LSDV), 10^7^ Colony Changing Units (CCU_50_) for CBPP and 10^4^TCID_50_ for Rift Valley fever virus (RVFV). The vaccines were reconstituted in PBS and injected subcutaneously (SC) in the neck. GII animals were vaccinated with overdoses of each vaccine, including a dose of 10^5.5^ TCID_50_ for LSDV, 10^8^ CCU_50_ for CBPP and 10^5^ TCID_50_ for RVFV. Vaccination with each vaccine was performed separately in the right side for LSD/CBPP vaccine and in the left side for RVF vaccine. Calves of GIII were kept as unvaccinated controls. Animals were monitored daily for two weeks post vaccination (pv) for the possible occurrence of local or general reactions. Body temperature was recorded two days before vaccination and daily for 14 days pv (dpv). The diameter of the local inflammation at the injection site was recorded daily for 2 weeks.

#### 2.3.3. Vaccination of Pregnant Females

As RVF is an abortive disease, the vaccine was tested in two- to three-year-old pregnant Holstein cows at different stages of pregnancy (2 to 8 months), which included 10 vaccinated animals (Group IV) and 8 unvaccinated animals (Group V) as negative controls (Table 1). The procedure for vaccinating pregnant cows was the same as described previously for LSD/CBPP+RVF vaccine with an overdose of 10^4.9^TCID_50_ for LSDV, 10^8^ CCU_50_ for CBPP and 10^5.5^ TCID_50_ for RVFV. Pregnant cows were kept under observation until calving and safety reproductive parameters (premature and full-term calving, abortion and physiological condition at birth). Newborn calves were examined daily during two weeks for general condition, presence of any malformation, body temperature and body weight.

### 2.4. Antibody Response following Vaccination

Fifteen cross-breed naïve Holstein calves, 4 to 6 months’ old, were randomly divided into 2 groups of 10 (GVI) and 5 (GVII) animals. All animals tested negative for antibody to the three vaccine pathogens using VNT and ELISA. Calves in GVI were injected with the LSD/CBPP+RVF vaccine as described for the GI animals, and GVII animals were not vaccinated. Blood samples for serological testing were collected in dry tubes via jugular venipuncture at weekly intervals throughout 42 dpv and thereafter at monthly intervals for 3 months pv. Sera were tested for antibody response to LSDV and RVFV using VNT and to CBPP using ELISA (IDEXX Ab Test, Montpellier, France). Serum samples were tested for specific RVFV and LSDV antibodies using a VNT as described in the WOAH Terrestrial Manual (Chapters 3.1.19 and 3.4.12). The VNT was performed by mixing equal volumes of serial 1/3 dilutions of heat-inactivated sera samples with 100 TCID_50_ of live virus (100 TCID_50_), and then inoculating aliquots of each dilution onto confluent monolayers of cells, and cells were observed once daily for 7 days for CPE. To validate the concentration of the viral dose, dilutions without sera were inoculated on cells and observed once daily for 7 days for CPE. The neutralizing antibody titer was calculated according to the method of Reed and Muench (1937) [42].

### 2.5. Vaccine Efficacy against RVFV and LSDV Challenges

The RVFV and LSDV challenge experiments were performed at the National Center for Foreign Animal Disease (Winnipeg, MB, Canada). Twelve 5- to 7-month-old calves seronegative to RVFV and LSDV were used in the experiments.

#### 2.5.1. RVFV Challenge

On D0, eight calves were injected with LSD/CBPP+RVF vaccine as described for the GI animals, and blood samples were obtained weekly for one month to test for VNT antibodies against RVFV and LSDV. On D28, vaccinated calves along with two unvaccinated animals were challenged by the intranasal route with 10^7^ pfu of the virulent strain of RVFV (Ken06). Animals were monitored for 21 days post infection (pi) for body temperature, clinical signs, neutralizing antibody and viral RNA in blood and nasal swabs using a quantitative real-time reverse transcriptase–polymerase chain reaction (RT-PCR) assay. Clinical scores based on signs of disease, body temperature, eating and drinking habits, disposition and stool consistency were used to evaluate the severity of clinical signs and to allow for a comparison between vaccinated and control animals. A total cumulative score was calculated based on the assessed clinical signs per animal per day. 

#### 2.5.2. LSDV Challenge

On D49 (D21 post RVFV infection), the same 10 challenged calves (8 vaccinated and 2 control), along with two other naïve calves, were each challenged via the intravenous route with one ml of 10^6^ pfu of the virulent strain of LSDV (Neethling). The animals were then monitored for three weeks to record body temperature and clinical signs, and to test serum samples for specific neutralizing antibodies and for viral DNA in the blood and nasal swabs using quantitative real-time PCR as previously described [43]. Three weeks pi, all calves were euthanized and examined for evidence of LSDV lesions. A clinical scoring was established based on general conditions, number and location of nodules, food uptake and lymph node swelling.

### 2.6. CBPP Challenge

The CBPP challenge was carried out at 6 months pv and at 13 months pv. The challenge experiments were subcontracted to Kenya Agricultural & Livestock Research Organization (KALRO), Nairobi, Kenya. Sixty 2- to 3-year-old naïve male cattle were purchased from a CBPP-free area in Kenya. The animals were already acclimatized and shown to be free of clinical signs of disease. The animals were randomized into 6 groups of 10 cattle each and then vaccinated with LSD/CBPP+RVF and commercial CBPP vaccines (Contavax), with saline used as a control (Table 2). Blood samples were obtained from the animals on D0, D14 and D28 pv, and then animals were subsequently challenged as reported in Table 2. The challenge was performed using a nebulizer model involving an aerosol-based intranasal technique where the cattle were infected via aerosols containing live *Mmm* (Afadé strain of CBPP) at 10^9^ Colony Forming Unit (CFU)/mL for 3 min, mimicking the infection of cattle in the field. This method provided a greater-than-80% efficiency in establishing infection in negative control cattle.

Challenged animals were monitored daily for clinical signs, body temperature and mortality. Blood was obtained from all animals on D7 before a challenge, and then on D14 and 28 pi. Necropsy was carried out 28 days pi and whenever an animal died during the study. Tissue sampling was performed by skilled veterinary pathologists to assess gross pathomorphological lesions, especially in the lung. Such lesions were measured and scored, and the severity was recorded. Sequestration, encapsulation, consolidation, fibrinous as well as fibrous adhesions, and their distribution between animals were noted.

The size of lung lesions was recorded in diameter, and lung pathology was scored according to the modified Hudson and Turner system (1963), in which the score was a combination of the size/type of the lesion and the isolation of the *Mmm* from tissues [44]. Lesions were also further described by whether there was a presence of fibrous/fibrinous adhesions, consolidation (hepatization) or sequestration, and this also informed the score. A lesion size < 5 was scored 1, lesions over 5 and under 20 were scored 2, and lesions > 20 were scored 3. In addition, if *Mmm* was isolated from the sample, a score of 2 was added to the total lesion score. Protection was calculated from the lesion indices of the control and vaccinated animals, again, according to Hudson and Turner, using the formula (NV – V) × 100/NV, where NV was the pathology index of the non-vaccinated group, and V is the pathology index of the vaccinated group.

### 2.7. Field Study

The LSD/CBPP+RVF vaccine was tested under field conditions to verify the safety and immunogenicity in cattle. The trial was conducted in four different provinces of Burkina Faso (Table 3). A total of 999 vaccinated cattle were distributed according to age: half-young (<1 year of age) and half adult (>1 year of age). The vaccinated cattle were observed for 42 dpv to detect a possible occurrence of abnormal local or general reactions. Blood samples were obtained from randomly sampled animals on D0 (before vaccination), D30 and D42 pv and tested for CBPP and RVF antibodies using ELISA (Idvet Innovative Diagnostics) using the immunoperoxidase monolayer assay (IPMA) for LSD antibodies as described by Haegeman et al. (2020) [45] (Table 3). Briefly, confluent 96-well plates containing Madin-Darby Bovine Kidney (MDBK) cells infected with 100 TCID_50_ of LSDV strain Neethling (LSDV-IPMA) were drained, dried after 3 days of incubation at 37 °C and frozen at −80 °C overnight. The cells were then fixed. The test samples were diluted 1:50 and 1:300 in blocking buffer, and each dilution was added in duplicate to the wells (50 µL/well) and incubated for 1 h at 37 °C. After emptying and washing the wells, horseradish peroxidase-conjugated antibody was added to each well. Plates were incubated at 37 °C for 1 h. After a final wash, substrate solution was added to each well to visualize the reaction. The mixture was incubated for 15 min at room temperature. The staining was stopped by removing the substrate and adding the Na-acetate buffer. Finally, the staining was analyzed using an inverted contrast microscope. In addition to the samples, one LSDV-positive and one LSDV-negative serum were added to each plate as controls and comparison points.

## 3. Results

### 3.1. Safety in Calves

**Figure 1:**
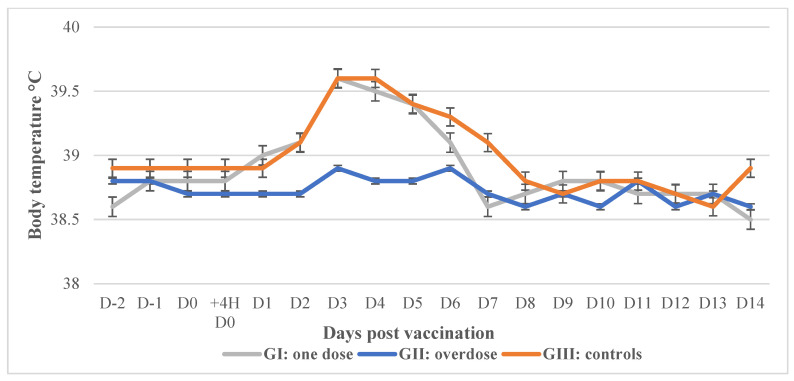
Average body temperature for each of the 2 groups that were vaccinated with the LSD/CBPP and RVF vaccine: 8 animals per group.

After the vaccination of each of the two groups of calves with the LSD/CBPP+RVF vaccine, the general body conditions remained normal during the observation period, and no cutaneous or respiratory symptoms were observed. Moderate hyperthermia was recorded in the vaccinated animals between 3 and 5 dpv (Figure 1). Limited local inflammations were recorded in 3/8 of vaccinated calves in GI and 4/8 of calves in GII (overdose) that evolve favorably. No cases of Neethling disease were observed in any vaccinated calves.

### 3.2. Safety in Pregnant Females

Among the vaccinated (with the LSD/CBPP and RVF vaccines) and unvaccinated pregnant cows, all animals remained healthy and did not show any signs of abortion or any abnormal reactions. All pregnant cows gave birth to healthy newborns calves with no malformation. During the follow-up period (14 days post parturition), the general health status of newborns was satisfactory for both vaccinated and unvaccinated cows (GIV and GV). No significant difference (*p* ≥ 0.05) was observed between the mean body weight and temperature of the newborns from the vaccinated and the control cows.

### 3.3. Immunogenicity Response

**Figure 2:**
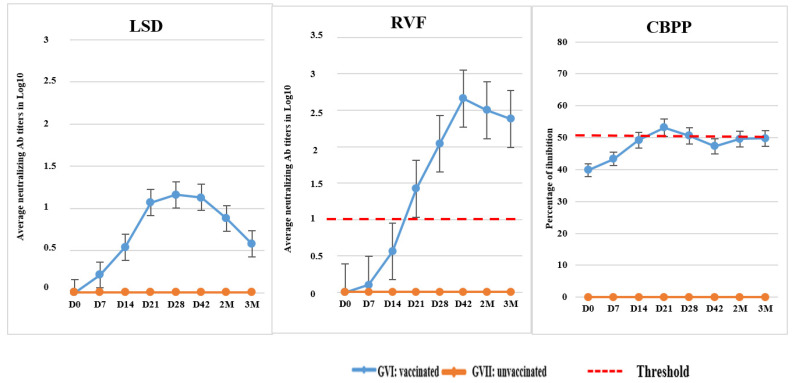
Average LSDV and RVFV antibody titers (VNT) and percentage of inhibition for CBPP (ELISA) in 10 calves of GVI vaccinated with LSD/CBPP+RVF vaccine and 5 unvaccinated animals of GVII. VNT titer >1.00 in log (equivalent to a serum dilution of 1/10) was considered positive for RVF. Percentage of inhibition > to 50% was considered positive for CBPP. D: Days; M: Months.

A neutralizing antibody against LSDV was detected in 2/10 calves on 7 dpv, and 6 of 10 calves seroconverted by 21 dpv. At 3 months pv, three calves remained positive (Table 4). The post-vaccination response to CBPP, as determined using ELISA, showed that one calf had seroconverted at 7 dpv, and 7 calves seroconverted by day 21 pv (Table 4). All animals seroconverted to the RVF vaccine between 7 and 28 dpv (Table 4 and Figure 2). No seroconversions were detected among the unvaccinated calves.

### 3.4. Efficacy of the Vaccine

#### 3.4.1. Challenging Calves with RVFV

Unvaccinated calves challenged with virulent RVFV showed evident hyperthermia between day 3 and day 5 pi reaching 41.4 °C. Nasal swabs were positive for viral RNA through real-time RT-PCR between D2 and D4 (Ct: 31.6) in one animal and from D1 to D10 (Ct: 33.3) in the other animal. Viremia was detected between D2 and D7 pi (peak at D3, Ct: 19.7) through both virus isolation and real-time RT-PCR as reported in Table 5.

**Figure 3:**
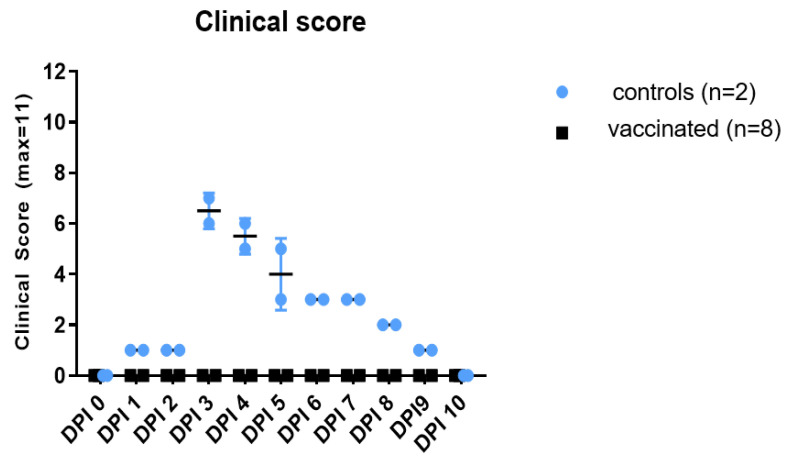
Clinical signs following RVFV challenge of 8 vaccinated and 2 unvaccinated calves.

In vaccinated calves, no clinical signs were reported during the observation period (Figure 3). Nasal swabs were negative after 2 dpi. In a few cases, there was some weak signal at 1 dpi, although this was either residual inoculum or beyond the limit of detection for the assay and was considered negative. No virus was isolated from any of the vaccinated animals. Vaccinated calves did not develop any detectable viremia through virus isolation or real-time RT-PCR, indicating that the vaccine was effective and prevented virus replication in the animals (Table 5).

**Figure 4:**
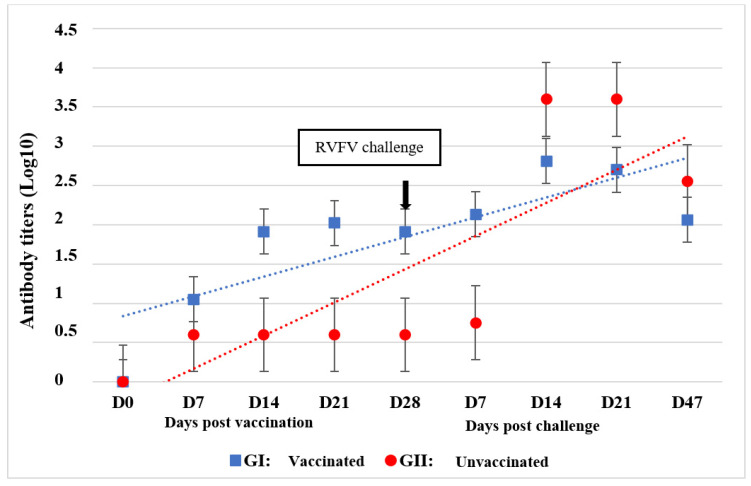
Mean neutralizing antibody titers to RVFV following vaccination and challenge of 8 vaccinated and 2 unvaccinated calves.

All RVF-vaccinated calves seroconverted to RVFV via VNT, and after being challenged, they developed an increase in neutralizing antibodies on day 14, with a decline on D47 pi (Figure 4).

#### 3.4.2. Challenging Calves with LSDV

**Figure 5:**
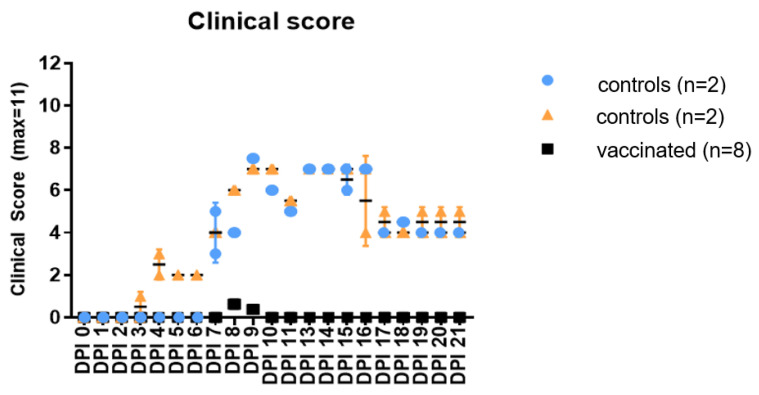
Clinical signs in 8 vaccinated and 4 unvaccinated calves following LSDV infection.

The four unvaccinated challenged calves exhibited fever (>40 °C) between D7 and D15 pi, reaching a temperature of 41.4 °C. Also, animals developed clinical signs of disease consisting of depression, reduced appetite and skin lumps, appearing 7 pi, which developed into skin lesions lasting until the end of the experiment (Figure 5). The animals shed viral DNA through real-time PCR in nasal swabs between D9 and D21 pi (Ct: 24–34), and viremia was detected through real-time PCR between 7 and 18 dpi (Ct: 30–35) (Table 6).

**Figure 6:**
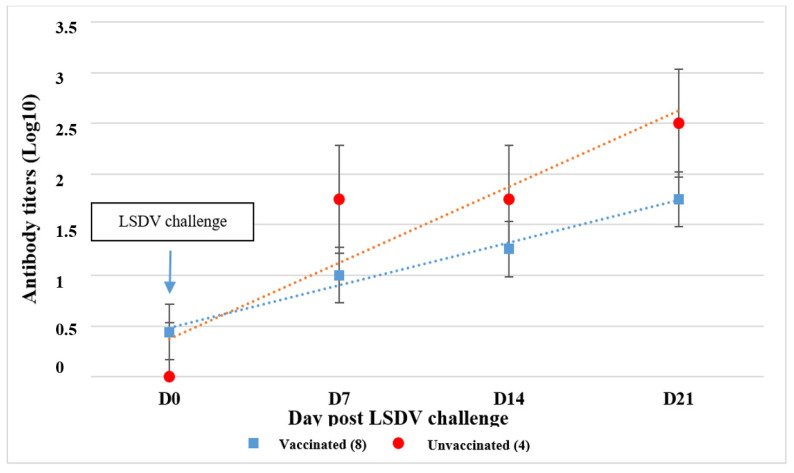
Mean neutralizing antibody titers among 8 vaccinated and 4 unvaccinated calves following LSDV challenge.

Clinical signs consisting of a mild depression and reduced appetite on 8 and 9 dpi were observed in some vaccinated calves, which were resolved by 10 dpi (Figure 5). All calves developed neutralizing antibodies, thus demonstrating that the animals were infected with LSDV (Figure 6). None of the animals developed a detectable viremia or viral shedding based on the testing of the nasal swabs using PCR, thus indicating that the vaccine was effective in preventing virus replication (Table 6).

#### 3.4.3. Challenging Calves with CBPP

##### CBPP Challenge at 6 Months pv

Regarding the challenge of the 10 unvaccinated control animals with CBPP, four animals developed moderate hyperthermia for 6 days, and lesions were observed in the lung of nine animals, eight of which were of high severity (Table 7). Among the animals vaccinated with the commercial vaccine, six developed hyperthermia during a 12-day period, and lesions were observed in the same six animals; four were severe. In animals vaccinated with the combined vaccine, two animals presented hyperthermia for a 6-day period, and moderate lesions were present in four animals, with severe lesions present in two of them (Table 7).

##### CBPP Challenge at 13 Months pv

Regarding the challenge of the unvaccinated animals with CBPP, 5 of 10 exhibited hyperthermia for 15 days, and 7/10 showed lesions of maximum severity. Among the challenged commercial vaccine group, 5 of 10 animals developed hyperthermia for 18 days. All animals of this group showed lesions in the lung; half of them were of high severity comparable with the unvaccinated control vaccine (Table 7).

Regarding the challenge of the 10 vaccinated calves with the combined LSD/CBPP+RVF vaccine, only 2 of them presented lesions and showed hyperthermia for 8 days. The lung lesions were severe in one animal and moderate in the second. The other eight animals did not develop a temperature or lesions (Table 7).

Using the pathology score, all unvaccinated challenged cattle were infected (Table 8). The protection rate in animals vaccinated with the combined vaccine and challenged had a protection rate of 50% at 6 months pv and 81% at 13 months pv (Table 8). In contrast, the protection rate for animals that received the commercial vaccine and challenged had a 38% protection rate at 3 months and 0% at 13 months.

### 3.5. Field Study

The combined LSD/CBPP+RVF vaccine was tested in cattle in a large-scale field study under natural conditions, and no abnormal reactions were observed among the vaccinated animals. Since the three diseases are enzootic in Africa, some of the animals used in the trial had antibodies as evidence of a previous infection on the vaccination day as shown in Table 9. However, after vaccination, there was a significant increase in the percentage of antibody-positive animals for the three vaccine pathogens.

## 4. Discussion

LSD, CBPP and RVF are enzootic diseases in large parts of the African continent that are economically important in the cattle production chain, causing high mortality, abortion and severe damage to hides [12,15,46,47]. Vaccination is the most appropriate tool to prevent infectious diseases, especially LSD and RVF, which are vector-borne diseases [19,39,48,49]. Multivalent vaccines present major benefits including low vaccination costs, higher vaccination coverage rate and less stress on the animals [28,30,50]. In addition, the inclusion of RVF will allow for vaccination against a disease considered as neglected in spite of its impact on public and animal health [46].

The formulation of two or more live attenuated pathogens as a multivalent vaccine can be a challenge since microorganism may interfere with and dominate the replication site [51]. This interference has been highlighted in previous experimental works on capripoxvirus and RVF [52]. Capripoxvirus infections are very common in large parts of Africa, Asia and the Middle East, and they affect both cattle and small ruminants [47,53]. These same animal populations are challenged by RVFV infection [54]. To address this possible interference, this study was conducted to evaluate the safety and potency of a combined LSD/CBPP and monovalent RVF freeze-dried vaccines to be injected separately and simultaneously into the same animals. Indeed, multivalent vaccines based on live attenuated microorganism are very rare in production animals [55,56]. In the market, live vaccines containing more than three organisms are absent, but this new LSD/CBPP+RVF formulation will allow for the vaccination of animals against three important diseases in one intervention. To our knowledge, this combination has never been tested before, although a bivalent LSD/CBPP vaccine has previously been tested with successful results and offers significant value to small-scale livestock keepers as a single administration [30].

In the present study, the LSD/CBPP+RVF vaccine was tested at the laboratory level for safety in young and pregnant females, antibody response to vaccination and efficacy through the challenge of the vaccinated animals with virulent pathogens. In addition, the vaccine was tested on a large number of animals in an area where these disease pathogens are enzootic [54,57]. The vaccine was completely safe, and vaccinated calves remained healthy with no excessive local swelling at the injection sites. In this trial, no abnormal local swelling that can be induced by CBPP, or LSD-like nodules that can attributed to Neethling vaccine strain commonly called Neethling disease, was observed [21,58,59]. These results confirmed the safety of the vaccine despite the limited number of animals tested at the laboratory level. RVFV is known to cause abortions in pregnant females, and any live attenuated vaccines should be safe for pregnant animals especially during the first stage of pregnancy [60,61]. In this experiment, no abortions or teratogenic effects were observed on the newborn calves, confirming that the vaccine has no negative effect on reproductive performance. This safety was confirmed in the field after the vaccination of cattle under different breeding conditions and health status.

The potency of the CBPP/LSD+RVF vaccine was evaluated based on the antibody response and protection against the challenge of the vaccinated animals with virulent pathogens. The antibody responses to the LSD vaccination was in accordance with observations obtained for the monovalent LSD vaccines, with the seroconversion rate being between 34% and 65%, as reported by Hamdi et al., 2020, Milovanović et al., 2019, and Samojlović et al., 2019 [17,62,63]. Concerning CBPP, the ELISA test was used to assess the antibody response as recommended by WOAH because of the higher sensitivity (96%) and specificity (97%) as compared to Complement Fixation Test (CFT) with sensitivity between 64% and 98% [10,13,64,65,66,67]. However, the antibody response assessed using ELISA or CFT did not correlate with protection and could not replace using a challenge [13]. In this experiment, antibody response reached 7/10 animals at 3 to 4 weeks pv as compared to 100% of seroconversion in the study conducted by Safini et al. 2021 [30]. However, the rate was higher compared to the rate reported by Nkando et al., 2012 (14–17% at 3 months pv), and Mwirigi et al., 2016 (37.5%), using monovalent live attenuated T1/ 44 vaccines [68,69]. Considering the RVFV antibody response in vaccinated animals, the results are in accordance with other studies that showed 100% seroconversion within 4 weeks pv [70,71,72]. This level of antibody suggests a full protection of vaccinated animals against RVFV, as several studies have shown that neutralizing antibodies was correlated with protection [60,73,74,75].

All unvaccinated animals, when challenged with the virulent strain of LSDV, showed typical symptoms of disease, which is not common for LSDV infections. The same challenge virus was previously used and resulted in variability in clinical signs of disease in susceptible cattle [76]. Meanwhile, 100% infection among control animals is rarely observed when challenged with LSDV; thus, 50 to 60% of infection in challenged unvaccinated controls may be sufficient to validate the protective efficacy test [77,78,79]. Based upon previously published data, it can be concluded that the challenge test using the intravenous route was successful and that this challenge model is robust and can be used to compare the different vaccines. The vaccinated animals showed full protection from the challenge at 7 weeks pv, which indicated that the required immunity may exceed one year pv. As reported in the study of Haegeman et al., 2023, the LSD attenuated live vaccine elicited a strong immune response and protection for up to 18 months [80]. In this study, all vaccinated animals were resistant to the challenge, and only two seroconverted, which indicated that cell-mediated immunity played a dominant role in capripoxvirus as reported by several authors [81,82].

Several factors were considered in the choice of an infection model for this challenge trial. In the absence of suitable laboratory animal models and in vitro methods, the vaccine conferred protection to CBPP was evaluated through the challenge of vaccinated cattle [83]. Existing challenge animal models for CBPP exhibited results ranging from no clinical disease to a wide spectrum of pathological lesions [26,84,85,86]. A common feature of these models was intubation that was required to reproduce the disease; it is necessary to use a large number of animals [83]. In-contact infection models were not considered a practicable alternative because it is difficult to synchronize the timing of individual infections and the observation of clinical signs. In addition, this model intrinsically contains more variations and requires a large number of animals [23,83]. Given the range of disease severity with this method, the development of a robust and reproducible challenge model for CBPP is clearly a priority [83,87].

In this study, the challenge test for infecting cattle with CBPP employed a nebulizer model, a method found (unpublished data) to provide a greater-than-80% efficiency in establishing infection in negative control cattle. The classical Hudson and Turner score was used to measure the protection rate, as it has frequently been used to ensure valid comparisons with former vaccine trials [88]. The performed test was valid since all unvaccinated challenged cattle were infected with a mean pathology score of 6.6 at 6 months and 4.2 at 13 months pv, scores suggesting a high intensity of the challenge and that the protection rates observed were very robust, as it is unlikely that animals in the field will be confronted with such a harsh challenge. The protection observed in vaccinated cattle challenged at 13 months was 81% higher than that observed at 6 months pv (50%). These results are in agreement with those of Wesonga et al., 2000, who reported an increase in protection from 59% at 3 months to 78.2% at 15 months [23]. Although efficacy rates were low at 6 months pv (50%), they were consistent with the results of previous studies that showed monovalent CBPP vaccines to elicit protection rates between 30% and 60% [18,89]. These rates were also within the WOAH recommendation (40–60%) for CBPP vaccines [10]. By testing two live vaccines through a challenge at 3 and 16 months, Nkando et al., 2012, reported a protection rate of 52–77% at 3 months pv and 56–62% at 16 months pv [68]. As reported, the monovalent vaccine requires booster vaccinations to achieve a 80–95% protection rate [23,58].

The development of reliable challenge models for arbovirus diseases like RVFV is challenging because needle inoculation does not mimic natural infection via insect vectors [90]. The common model for RVFV is pregnant ewe because abortions are a hallmark among livestock [91]. However, pregnancy synchronization in ewes and the limited high biosecurity and biocontainment of animal spaces makes this model difficult to use. In previous RVF challenge models, the SC route has been used, and this method provides fever and viremia in inoculated animals [90,92]. In this study, the intra-nasal route was used to challenge cattle, as tested in previous studies, and it elicited viremia and fever sufficient to evaluate vaccines [93]. Using this challenge model, all vaccinated animals were protected, and the unvaccinated animals developed a fever, viral shedding and a viremia. This confirms that there was a positive correlation between protection and neutralizing antibodies [60,94].

The multivalent LSD/CBPP+RVF vaccine was tested in a sufficient number of animals under field conditions, with no adverse effects pv. The seroconversion rate increased after vaccination for the three diseases, despite being low for CBPP and RVFV, as compared to laboratory results, probably because the animals were taken at random and antibody response monitoring was not carried out in the same animals. A field study was conducted with the inactivated LSD vaccine and showed that 70% of the animals developed neutralizing antibodies when VNT was used [62]. Other studies have reported seroconversions of 85.15% of animals vaccinated with LSD live attenuated vaccine on day 30 pv [95]. Regarding CBPP, there is a need to have tests that can provide sensitive and specific outcomes in the field [13]. Concerning RVF, a field study using RVF C13T vaccine was conducted in Tanzania and showed a seroconversion rate of 57.1% in vaccinated cattle on day 30 pv compared to the rate of 56.6% observed in our study [96]. This observation is in contrast to the findings from studies in Kenya that reported that vaccinating cattle with RVFV C13T failed to develop RVFV IgM antibody [71], as well as a study in Senegal that found a very low proportion of IgM seropositivity among vaccinated sheep and goats [97].

## 5. Conclusions

This study is the first report on the evaluation of a vaccine consisting of the combination of three live pathogens, including Mycoplasma CBPP T1 44 attenuated strain, LSD Neethling attenuated strain and RVF C13 T strain. The findings revealed that the simultaneous vaccination of the animals with the combined vaccine afforded protection to cattle against challenge by virulent etiological agents of the three diseases. Only one vaccination was sufficient to induce at least 12 months of protection, as demonstrated in this study via the protection of cattle against CBPP at 13 months pv and via the protective antibody responses to RVF and LSD diseases.

This LSD/CBPP+RVF vaccine will benefit many countries in Africa and the Middle East where the three diseases coexist because one vaccination will afford protection against three highly important diseases of cattle. The vaccine is safe and efficacious, and it will reduce the vaccination cost, improve vaccination coverage and enable the implementation of regular RVF vaccination. 

## Figures and Tables

**Table 1:** Vaccination of 10 cows at different stages of pregnancy (combined LSD/CBPP+RVF at a dose of 10^4.9^ TCID_50_ for LSDV, 10^8^ CCU_50_ for CBPP and 10^5.5^ TCID_50_ for RVFV).

Stage of Gestation/Group	2M	3M	4M	5M	6M	7M	8M
GIV: 10 vaccinated cows	1	1	0	2	0	4	2
GV: 8 controls cows	1	1	1	1	1	3	0

**Table 2:** Number of cattle randomized into 6 groups of 10 cattle each for vaccination with respective vaccines and challenges of the vaccinated animals and controls with the virulent strain of CBPP.

Group	Vaccine	No. of Cattle	Challenge Phase pv
1	LSD/CBPP+RVF	10	6 months
2	LSD/CBPP+RVF	10	13 months
3	Commercial CBPP vaccine (Contavax)	10	3 months
4	Commercial CBPP vaccine (Contavax)	10	13 months
5	Saline	10	6 months
6	Saline	10	13 months

**Table 3:** Number of cattle and province involved in the field vaccine trial of the LSD/CBPP and RVF vaccines and number of cattle sampled in each phase on D0, D30 and D42 post vaccination.

Province	Climatic Zone	Number of Vaccinated Animals	Blood Sample/Phase		
			D0	D30	D42
Ziro	Sudanese	200	19	19	19
Tuy	Sudanese	200	20	20	20
Sanmatenga	Sub-Sahel	450	47	43	44
Bazéga	North Soudanese	149	27	24	29
**Total**		**999**	**113**	**106**	**112**

**Table 4:** Number of antibody and ELISA-positive calves after vaccination of 10 calves with LSD/CBPP+RVF vaccine. The 10 animals were tested using VNT (LSDV and RVFV) and ELISA (CBPP).

Post Vaccination	D0	D7	D14	D21	D28	D42	2M	3M
**LSDV**	0	2	3	6	5	5	4	3
**CBPP**	0	1	5	7	7	4	4	3
**RVFV**	0	1	5	9	10	10	10	10

**Table 5:** RVFV viral RNA detected in sera of 8 vaccinated (all are negative for RNA, only in unvaccinated animals) and 2 unvaccinated calves through real-time RT-PCR after challenge.

Group	Calves	Days Post Challenge with RVFV							
		1	2	3	4	7	10	14	21
**Vaccinated**	307	0	0	0	0	0	0	0	0
	310	0	0	0	0	0	0	0	0
	311	0	0	0	0	0	0	0	0
	2076	0	0	0	0	0	0	0	0
	2453	0	0	0	0	0	0	0	0
	2454	0	0	0	0	0	0	0	0
	2456	0	0	0	0	0	0	0	0
	2457	0	0	0	0	0	0	0	0
**Unvaccinated**	306	0	30.0	23.8	25.8	39.0	0	0	0
	2086	0	27.3	19.7	26.7	38.0	0	0	37.0

**Table 6:** LSDV viral DNA detected in nasal swabs of 8 vaccinated and 4 unvaccinated calves through real-time PCR following challenge with virulent LSDV.

Group	Calves	Days Post Challenge with LSDV							
		1	2	3	4	7	10	14	21
**Vaccinated**	307	-	-	-	-	-	-	-	-
	310	-	-	-	-	-	-	-	-
	311	-	-	-	-	-	-	-	-
	2076	-	-	-	-	-	-	-	-
	2453	-	-	-	-	-	-	-	-
	2454	-	-	-	-	-	-	-	-
	2456	-	-	-	-	-	-	-	-
	2457	-	-	-	-	-	-	-	-
**Unvaccinated**	2458	-	-	-	33	34	32	31	30
	246-3	-	-	-	-	27	31	33	-
	306	-	-	28	27	24	32	30	29
	2086	-	-	-	32	29	26	32	34

**Table 7:** Number of days of fever and lung lesions in cattle after CBPP challenge. A total of 10 cattle per group in each challenge phase.

	Challenge 6 Months			Challenge 13 Months		
Group	Hyperthermia Duration (Days)	Number of Animals Presenting Lesions	Severity Lesions	Hyperthermia Duration (Days)	Number of Animals Presenting Lesions	Severity Lesions
**Saline**	6	9	8	15	7	7
**Commercial**	12	6	4	18	10	5
**Multivalent LSD/CBPP+RVF**	6	6	2	8	2	1

**Table 8:** Calculation of protection efficacy for each group of cattle challenged with CBPP strain. A total of 10 cattle per group in each challenge phase.

	Protective Efficacy Calculations 6 Months				Protective Efficacy Calculations 13 Months			
Group	Mean Total Score	Vaccination/Control Ratio	Efficacy: (1-Ratio)	% Protection	Mean Total Score	Vaccination/Control Ratio	Efficacy: (1-Ratio)	% Protection
**Saline**	6.6	1	0	**0%**	4.2	1	0	**0%**
**Commercial vaccine**	4.1	0.62	0.38	**38%**	4.2	1	0	**0%**
**Multivalent LSD/CBPP+RVF**	3.3	0.5	0.5	**50%**	0.8	0.2	0.8	**81%**

**Table 9:** Percentage of antibody-positive cattle vaccinated with combined LSD/CBPP+RVF vaccine during a large-scale field trial; sera samples were obtained from the cattle on days 0, 30 and 42 and tested using ELISA for CBPP/RVFV and IPMA-LSDV antibodies.

	Percentage of Positive Animals		
Valence/Phase pv	D0	D30	D42
**LSDV**	41.5%	50.0%	43.7%
	(47/113)	(53/106)	(49/112)
**CBPP**	19.4%	43.3%	44.6%
	(22/113)	(46/106)	(50/112)
**RVFV**	21.2%	56.6%	41.0%
	(24/113)	(60/106)	(46/112)

## Data Availability

The datasets used and/or analyzed during the current study are available from the corresponding author on reasonable request.
